# Complexity of Heart Rate Variability Can Predict Stroke-In-Evolution in Acute Ischemic Stroke Patients

**DOI:** 10.1038/srep17552

**Published:** 2015-12-01

**Authors:** Chih-Hao Chen, Pei-Wen Huang, Sung-Chun Tang, Jiann-Shing Shieh, Dar-Ming Lai, An-Yu Wu, Jiann-Shing Jeng

**Affiliations:** 1 Stroke Center and Department of Neurology, National Taiwan University Hospital, Taipei, Taiwan; 2 Division of Neurology, Department of Internal Medicine, Far-Eastern Memorial Hospital, New Taipei, Taiwan; 3 Graduate Institute of Epidemiology and Preventive Medicine, National Taiwan University, Taipei, Taiwan; 4 Graduate Institute of Electronics Engineering, National Taiwan University, Taipei, Taiwan; 5 NTU-NTUH-MediaTek Innovative Medical Electronics Research Center, Taipei, Taiwan; 6 Department of Mechanical Engineering and Innovation Center for Big Data and Digital Convergence, Yuan Ze University, Taoyuan, Taiwan; 7 Division of Neurosurgery, Department of Surgery, National Taiwan University Hospital, Taipei, Taiwan

## Abstract

About one-third of acute stroke patients may experience stroke-in-evolution, which is often associated with a worse outcome. Recently, we showed that multiscale entropy (MSE), a non-linear method for analysis of heart rate variability (HRV), is an early outcome predictor in non-atrial fibrillation (non-AF) stroke patients. We aimed to further investigate MSE as a predictor of SIE. We included 90 non-AF ischemic stroke patients admitted to the intensive care unit (ICU). Nineteen (21.1%) patients met the criteria of SIE, which was defined as an increase in the National Institutes of Health Stroke Scale score of ≥2 points within 3 days of admission. The MSE of HRV was analyzed from 1-hour continuous ECG signals during the first 24 hours of admission. The complexity index was defined as the area under the MSE curve. Compared with patients without SIE, those with SIE had a significantly lower complexity index value (21.3 ± 8.5 vs 26.5 ± 7.7, *P* = 0.012). After adjustment for clinical variables, patients with higher complexity index values were significantly less likely to have SIE (odds ratio = 0.897, 95% confidence interval 0.818–0.983, *P* = 0.020). In summary, early assessment of HRV by MSE can be a potential predictor of SIE in ICU-admitted non-AF ischemic stroke patients.

Approximately one-third of patients with acute ischemic stroke may suffer from early worsening of neurological symptoms, or stroke-in-evolution, which is often associated with poor clinical outcome^1^,^2^,^3^,^4^. Therefore, early identification of SIE is crucial in acute stroke management. On the other hand, stroke commonly affects the autonomic nervous system and induces cardiovascular responses^5^,^6^. Studies have shown that a reduction of heart rate variability (HRV) is an indicator of general illness, including acute stroke^7^. These studies usually applied conventional linear HRV analyses such as frequency and time domain analyses, and their prognostic values for acute stroke were inconsistent^7^,^8^,^9^,^10^. The modulation of HRV, however, is thought to be originated from non-linear processes with non-stationary property^11^,^12^. Applying linear algorithms to these seemingly irregular cardiovascular signals may cause intrinsic computational errors of the linear algorithms^13^,^14^,^15^. Properly use of the analyses based on chaos theory to better describe the characteristics of heart rate time series are a more reliable index of physiological systems in many clinical studies^13^,^16^. Recently, multiscale entropy (MSE) has been developed as a non-linear method to quantify the complex regulatory dynamics of human biological signals, such as HRV^11^. We have shown that early assessment of HRV by MSE can help predict outcomes in patients with non-atrial fibrillation (non-AF) stroke^17^. The present study aimed to further investigate whether MSE is a predictor of SIE in non-AF ischemic stroke patients.

## Results

During the study period, 384 consecutive acute stroke patients admitted to the stroke intensive care unit (ICU) received electrocardiography (ECG) monitoring and HRV analyses. We excluded 120 patients with intracerebral hemorrhage, 120 patients with AF rhythm, and 6 patients without a complete record of SIE. Because we intended to investigate the early predictive ability of MSE analysis, we further excluded 48 patients who did not receive MSE until 24–48 hours after admission. A total of 90 patients with non-AF acute ischemic stroke were finally included in the analysis, and 57 (63.3%) of them had anterior circulation stroke. Nineteen (21.1%) patients met the criteria for SIE, and they had a significantly higher median National Institute of Health Stroke Scale (NIHSS) score at admission (17 vs 10, *P* = 0.048), and a lower percentage had hypertension, diabetes mellitus, and history of smoking, and they had a lower initial blood glucose level (Table 1). Of the linear parameters of HRV, only root-mean-square of successive beat-to-beat differences (RMSSD) and the low-frequency to high-frequency (LF-HF) ratio were significantly different between patients with and without SIE (*P* = 0.030 and 0.018, respectively). Meanwhile, the MSE values were significantly lower in patients with SIE, including the complexity index (21.3 ± 8.5 vs 26.5 ± 7.7, *P* = 0.012) and Area_6-20_ (16.0 ± 6.2 vs 20.5 ± 6.2, *P* = 0.006).

**Figure 1:**
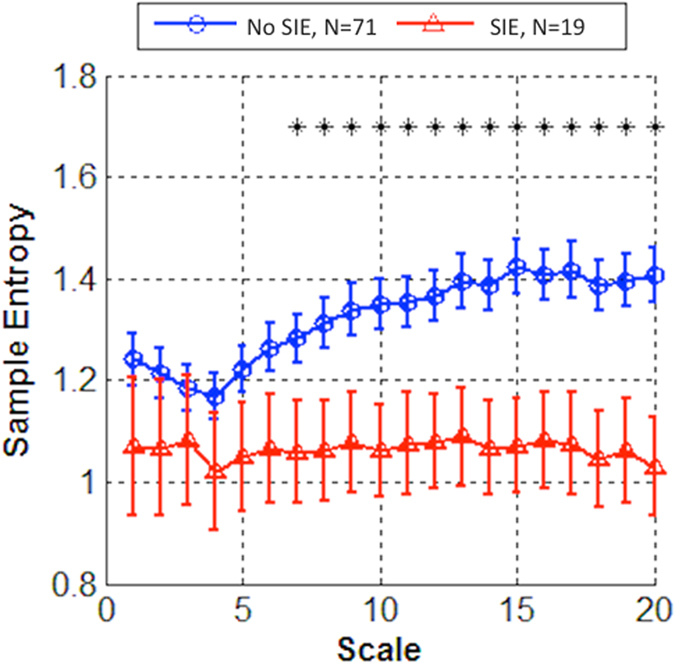
Multiscale entropy (MSE) curves enable to differentiate among patients with and without stroke-in-evolution (SIE). In patients with stroke-in-evolution, the MSE curves were apparently lower especially in time scales 6–20.

In the multivariable logistic regression model after adjustment for age, sex, history of hypertension, history of smoking, NIHSS score and glucose level at admission, only the MSE-derived measurements were significant predictors for SIE among the various parameters of HRV (Table 2). Patients with higher values of complexity index (OR 0.897, *P* = 0.020), Area_1-5_ (OR 0.715, *P* = 0.046) or Area_6-20_ (OR 0.868, *P* = 0.020) were less likely to have SIE. The values of RMSSD and LF-HF ratio did not have significant associations with SIE (OR 1.014, *P* = 0.058 and OR 0.514, P = 0.080, respectively). The Fig. 1 shows the plotted MSE curves in patients with and without SIE. The sample entropies were apparently lower in patients with SIE than those without SIE, and the curves were more discriminative in scales 6–20.

## Discussion

In the present study, we found that a lower complexity of HRV, assessed by MSE, is related to the occurrence of SIE. In patients with acute ischemic stroke, the major causes of SIE include new or progressive stroke, vasogenic edema, raised intracranial pressure, hemorrhagic transformation of infarcts, and seizure^1^,^2^. Many predictors of SIE have been proposed in previous studies. According to a recent review, the strongest and most consistent admission predictors are hyperglycemia, no prior aspirin use, prior transient ischemic attacks, proximal arterial occlusion, and presence of early CT changes^1^. However, most predictors are related to the pre-stroke background information rather than post-stroke brain injury. Since acute stroke commonly induces change in cardiovascular responses^5^,^6^, post-stroke BP and HR at admission could be potential markers to identify patients at risk for short-term deterioration or worse long-term outcome. However, simple cut-off values for BP or HR data, or conventional HRV analyses to predict stroke outcome, may not reflect the true influences and complexity of the autonomic nervous system, resulting in inconsistent findings from previous studies^6^,^7^,^8^,^9^,^10^.

The novel non-linear method of MSE can be used to calculate entropy over multiple time scales and enables the evaluation of the information richness in HRV. Decreased complexity of HRV assessed by MSE has been demonstrated in normal aging people and in patients with type 1 diabetes mellitus, congestive heart failure, and acute stroke with poor outcome^11^,^17^,^18^,^19^. Our study clearly revealed that MSE analysis is superior to conventional linear analyses of HRV for predicting SIE. The summation of entropy values over different time scales may reflect certain behavior of heart rate dynamics. As shown in the Figure, the differences of the sample entropy values were more apparent for longer time scales. In our study, the Area_1-5_ showed borderline associations with SIE (*P* = 0.046), while Area_1-20_ and Area_6-20_ were stronger predictors. A previous study also identified Area_6-20_ as a predictor of mortality in patients with heart failure^18^. It is possible that the complexity of HRV at the longer time scale more closely reflects the underlying systemic hemodynamic turbulences. Since patients admitted to the ICU were routinely monitored by bedside ECG, and MSE analysis only required 1-hour ECG data, our results imply that this method might be applicable in acute stroke management.

Our study had some limitations. First, the sample size was relatively small and our patients were from a single hospital. We only included acute ischemic stroke patients with non-AF rhythm, the generalizability of our results might be limited. Second, we did not specifically separate out strokes with insular cortex involvement to test whether HRV was uniquely affected in this group of patients, as has been demonstrated by many previous works^5^,^6^. However, we did not find stroke locations (either anterior vs posterior circulation, or right vs left hemisphere) as predictors of SIE (data not shown). Third, because our previous study had shown that the value of the complexity index provided no outcome information in patients with AF stroke, we excluded this group from our analysis. Further efforts to develop appropriate mathematical algorithms in patients with AF rhythm are warranted.

In conclusion, our single center study found that acute ischemic stroke patients suffering from SIE had a significant reduced complexity of HRV. Early assessment of HRV by the non-linear MSE method can be a potential predictor of SIE in ICU-admitted non-AF ischemic stroke patients, and the finding should be confirmed in a larger study population in the future.

## Methods

### Study design, Setting, and Patients

This study, including the data and experimental protocols, were approved by the Institutional Review Board of National Taiwan University Hospital to prospectively collect information on acute stroke patients, including stroke severity, risk factors, stroke mechanism, and outcome. All patients gave their written informed consent. The methods were carried out in accordance with the approved guidelines.

Patients with acute stroke admitted within 24 hours to the stroke ICU from January 2012 to June 2014 were studied prospectively. The admission criteria for the stroke ICU included receiving thrombolytic therapy or endovascular treatment, intracerebral hemorrhage receiving aggressive blood pressure control, severe neurological deficits (e.g., NIHSS score >8), stroke-in-evolution, or medical conditions requiring intensive care. The diagnosis of acute ischemic stroke was confirmed by computed tomography of the head or magnetic resonance imaging, and stroke locations were classified as anterior or posterior circulation territories. All patients received standard intensive care. Vital sign monitoring included blood pressure, heart rate, ECG, respiratory rate, and oxygen saturation. Patients with modified Rankin scale >2 prior to the index event, symptomatic heart failure, inability to obtain ECG signals within 48 hours after admission, and poor quality or artifacts of ECG signals were excluded from analysis.

Stroke severity was categorized based on the admission NIHSS score. NIHSS assessment was routinely repeated at 24 hours after admission, and under any circumstance when significant clinical change was suspected. SIE was defined as an increase in the NIHSS score of ≥2 points within 3 days of admission, excluding other attributable medical or systemic causes.

### ECG Data Acquisition and Analysis

Details of the method used for ECG data recording and analysis have been previously published^8^. Briefly, we collected ECG analogue data directly from the stroke ICU bedside monitor (Philips Intellivue MP70) for each patient. We collected the ECG data as soon as possible upon patient’s admission to the stroke ICU. To prevent the possible bias of data selection, only the first hour of the data was taken for analysis in all study subjects. One-hour ECG data were digitized with a sampling rate of 512 Hz and stored in a computer. The stored ECG data then went through a pre-processing step to extract the R-R interval time series before analysis. Atrial fibrillation rhythm was indicated as the absence of P waves with irregular R-R intervals.

Linear analysis of HRV included time-domain and frequency-domain measures. The two time-domain analyses were standard deviation of normal to normal R wave (SDNN) and root-mean-square of successive beat-to-beat differences (RMSSD). The frequency-domain HRV analysis included high frequency power (0.15–0.4 Hz), low-frequency power (0.04–0.15 Hz), and the ratio of low-frequency to high-frequency (LF-HF) power.

Non-linear MSE analysis was applied to the acquired 1-hour ECG data and it comprised two steps: (1) coarse-graining the signals into different time scales, (2) sample entropy calculation for each coarse-grained time series to quantify the degree of irregularity^12^. Then the entropy is calculated as a function of scale to provide a measure of information richness embedded in different time scales. We coarse-grained the original time series up to a scale factor of 20, which is a typical choice in most studies^12^,^17^,^18^. A detrending process was performed to attenuate the influence caused by non-stationary artifacts^14^. We calculated three different parameters derived from the MSE profile: the summations of quantitative values of scale 1–5 (Area_1–5_), scale 6–20 (Area_6–20_), and all scales (Area_1–20_, named the ‘complexity index’), which represented the short-term, long-term, and overall complexity, respectively^18^. Our previous study showed that MSE analysis had poor predictive ability on outcome of patients with AF^17^. Hence, we only included patients with non-AF rhythm into our analysis.

### Statistical Analysis

The clinical characteristics, linear parameters of HRV, and MSE values for patients with and without SIE were compared using χ^2^ test, Fisher’s exact test, Student *t* test, and Mann-Whitney U test with relevant variables as indicated. We performed multivariable logistic regression models that were adjusted for age, sex, initial NIHSS score, along with other significant or relevant variables, to test for an association between the presence of SIE and MSE value (MSE value entered as a continuous variable). MSE curves were plotted and complexity index values were presented as the mean ± standard deviation. A *P* value of <0.05 was considered to indicate statistical significance. The SAS version 9.3 (SAS Institute Inc, Cary, NC, USA) was used for all analyses.

## Additional Information

**How to cite this article**: Chen, C.-H. *et al.* Complexity of Heart Rate Variability Can Predict Stroke-In-Evolution in Acute Ischemic Stroke Patients. *Sci. Rep.* **5**, 17552; doi: 10.1038/srep17552 (2015).

## Figures and Tables

**Table 1:** Variables of the study population related to the occurrence of stroke-in-evolution.

	No SIE (n = 71)	SIE (n = 19)	*P* value
Age, years	64.0 ± 15.3	67.2 ± 18.2	0.45
Male	39 (54.9)	9 (47.4)	0.56
Hypertension	57 (80.3)	17 (52.6)	0.01
Diabetes mellitus	34 (47.9)	3 (15.8)	0.01
Dyslipidemia	41 (57.8)	7 (36.8)	0.10
Prior stroke	21 (29.6)	3 (15.8)	0.18
Smoking	28 (39.4)	2 (10.5)	0.01
Glucose, mg/dL	160.2 ± 78.3	123.8 ± 29.5	0.003
NIHSS at admission	10 (5–18)	17 (8–22)	0.05
Anterior circulation stroke	45 (64.3)	12 (63.2)	0.93
Hear rate variability parameters			
SDNN	57.4 ± 37.4	82.1 ± 59.9	0.10
RMSSD	45.2 ± 34.8	92.0 ± 85.1	0.03
High frequency	401.6 ± 661.9	1592.1 ± 3535.3	0.16
Low frequency	397.6 ± 352.1	822.7 ± 1473.4	0.23
LF-HF ratio	1.92 ± 1.10	1.26 ± 0.93	0.018
Area_1-5_	6.03 ± 1.90	5.29 ± 2.62	0.17
Area_6-20_	20.5 ± 6.2	16.0 ± 6.2	0.006
Complexity index (Area_1-20_)	26.5 ± 7.7	21.3 ± 8.5	0.01

Data are expressed as mean ± standard deviation or n (%), except NIHSS is median (interquartile range). NIHSS: National Institutes of Health Stroke Scale, SDNN: standard deviation of normal to normal R wave, RMSSD: root-mean-square of successive beat-to-beat differences, LF-HF: low-frequency to high-frequency.

**Table 2:** Adjusted odds ratio (OR) of parameters in predicting stroke-in-evolution.

	Adjusted OR (95% CI)*	*P* value
SDNN	1.009 (0.995–1.023)	0.201
RMSSD	1.014 (1.000–1.028)	0.054
High frequency	1.000 (1.000–1.001)	0.328
Low frequency	1.000 (1.000–1.001)	0.374
LF-HF ratio	0.514 (0.245–1.082)	0.080
Area_1-5_	0.715 (0.514–0.994)	0.046
Area_6-20_	0.868 (0.770–0.978)	0.020
Complexity index (Area_1-20_)	0.897 (0.818–0.983)	0.020

CI: confidence interval, SDNN: standard deviation of normal to normal R wave, RMSSD: root-mean-square of successive beat-to-beat differences, LF-HF: low-frequency to high-frequency. * adjusted for age, sex, hypertension, smoking, NIHSS score and blood glucose level at admission.
